# Persistence of OXA-48-producing ST-22 *Citrobacter freundii* in patients and the hospital environment, Paris, France, 2016 to 2022

**DOI:** 10.2807/1560-7917.ES.2024.29.49.2400262

**Published:** 2024-12-05

**Authors:** Sarah Jolivet, Jeanne Couturier, Killian Le Neindre, Muriel Ehmig, Laurent Dortet, Cécile Emeraud, Frédéric Barbut

**Affiliations:** 1 Unité de prévention du risque infectieux, Hôpital Saint-Antoine, Assistance Publique - Hôpitaux de Paris, Paris, France; 2 Service de microbiologie de l’environnement, Hôpital Saint-Antoine, Assistance Publique - Hôpitaux de Paris, Paris, France; 3 INSERM 1139, 3PHM, Université de Paris Cité, Paris, France; 4 Associated French National Reference Center for Antibiotic Resistance: Carbapenemase-producing Enterobacterales, Le Kremlin-Bicêtre, France

**Keywords:** carbapenemase-producing Enterobacterales, *Citrobacter freundii*, reservoir, whole genome sequencing, water environment

## Abstract

In 2016–2019, hospital A’s haematology ward experienced an outbreak of OXA-48-producing ST-22 *Citrobacter freundii* strains, with toilets identified as source of transmission. Between 2020 and 2022, 28 strains of OXA-48-producing ST-22 *C. freundii* were isolated on other wards. This study aimed to determine whether all OXA-48-producing ST-22 *C. freundii* strains belonged to the same clone and to investigate the persistence of this clone using whole genome sequencing. OXA-48-producing ST-22 *C. freundii* strains collected from patients (n = 33) and from the hospital environment (n = 20) of seven wards were sequenced using Illumina technology and clonal relationships were determined using single nucleotide polymorphism (SNP). Phylogenetic analyses were performed on 53 strains from hospital A and on 240 epidemiologically unrelated carbapenem-resistant ST-22 *C. freundii* isolated from elsewhere in France. SNP analysis suggested long-lasting persistence of the same clone for more than 6 years. Phylogenetic analysis showed that 52 of 53 strains isolated in hospital A belonged to the same cluster and were different from the 240 epidemiologically unrelated *C. freundii* ST-22. Our data suggest that this clone can persist in hospital environments for years, representing a risk for hospital-acquired infections and outbreaks. Reservoir management is essential to prevent further transmission.

**Key public health message**

**What did you want to address in this study and why?**

Carbapenems are effective antibiotics used to treat serious and multidrug-resistant bacterial infections. We investigated the spread and the persistence of highly carbapenem-resistant bacteria, *Citrobacter freundii* (*C. freundii* ST-22) that produces carbapenamase OXA-48 and belongs to the group Enterobacterales, obtained from patients and the environment in a hospital in France between 2016 and 2022.

**What have we learnt from this study?**

All bacteria strains obtained from patients and the hospital environment (toilets and drains) were closely related. This strain can persist in the environment for several years and spread through buildings, representing a risk of hospital-acquired infections and outbreaks in hospitalised patients.

**What are the implications of your findings for public health?**

Managing environmental contamination is essential to prevent further transmission and long-term outbreaks in hospitals. Our findings highlight the need for epidemiological and environmental investigations to manage resistant Enterobacterales outbreaks in hospitals.

## Background

The incidence of carbapenemase-producing Enterobacterales (CPE) is steadily increasing worldwide and in Europe [1]. Carbapenemase-producing Enterobacterales infections can lead to treatment failure, extended hospital stays, increased healthcare costs and increased mortality [2-5]. *Citrobacter freundii* is found in the environment (soil, wastewater) and is a commensal bacterium in the digestive tract [6,7]. These bacteria are opportunistic pathogens able to induce a large range of infections such as urinary tract infections, meningitis, respiratory tract infections, gastrointestinal infections or neonatal sepsis particularly in high-risk populations such as immunocompromised patients [8,9]. They are also frequently associated with hospital outbreaks, the number of which has increased in the past few years [8,10,11]. Whole genome sequencing (WGS) has been successfully used to trace transmission chains and reveal unexpected transmission routes during outbreaks [12].

Hospital sanitary installations (drains, sinks and water taps) have been identified as potential CPE reservoirs [13]. These reservoirs have been linked to long-term transmissions and outbreaks [14]. Management of these reservoirs with cleaning and disinfection is often challenging because of the persistence of this pathogen [15].

## Outbreak detection

The haematology ward of our hospital (hospital A) experienced a large outbreak of OXA-48 CPE, mostly OXA-48-producing *C. freundii* sequence type ST-22, between 2016 and 2019 [16]. The outbreak was successfully controlled only after replacing all toilets, which were recognised as the source of transmission. Two years later, OXA-48-producing ST-22 *C. freundii* was isolated in 15 patients in five other wards located in different buildings of hospital A between 2020 and 2022, with no obvious link with the haematology ward.

In this study, we investigated the spread and persistence of an OXA-48-producing ST-22 *C. freundii* population in hospital A between 2016 and 2022 using WGS and phylogenetic analyses.

## Methods

### Setting and design

Hospital A is a 673-bed teaching, acute care hospital in Paris, France. It includes 17 clinical wards located in seven different buildings. In 2022, the hospital recorded 23,156 admissions resulting in a total of 186,525 hospital bed-days.

We conducted a retrospective analysis of bacterial strains because of an unexpected high proportion of OXA-48-producing ST-22 *C. freundii* between 2016 and 2022.

### Case definitions

Cases were defined as patients colonised or infected by OXA-48-producing ST-22 *C. freundii* between January 2016 and July 2022.

The strain was considered as acquired if the patient tested positive after 48 hours from admission, even if no screening had taken place at admission. The strain was considered as imported if the patient tested positive in the first 48 hours from admission. A patient was considered as simply colonised if the strain was only isolated from a rectal swab and infected if the strain was isolated from clinical samples (blood, urine, respiratory sample).

### Infection control measures

Infection control measures for colonised or infected patients were implemented according to the French national guidelines [17,18]. They consisted of strict contact precautions in a single bedroom and a screening of contact patients. A contact patient was defined as any patient cared for by the same healthcare team as a CPE case.

In hospital A, patients are screened for CPE carriage by rectal sample if they have any history of hospitalisation or haemodialysis abroad (whatever the country) in the previous year. Since 2020, patients who have travelled abroad within 3 months before hospital admission are also screened. In addition, from January 2016 to July 2022 patients hospitalised in the surgical intensive care unit or haematology ward were systematically screened on admission and weekly until discharge from these units. Furthermore, additional weekly screening was conducted for contact patients for as long as the CPE index case was hospitalised on a given ward, and at least once after the case was discharged. Rectal samples were collected with an eSwab system (COPAN, Brescia, Italy).

### Environmental investigation

Between January 2016 and July 2022, environmental sampling was performed to identify potential CPE reservoirs after room cleaning following the discharge of a CPE carrier or during outbreak investigations. Sampling was performed only upon infection control practitioner request to explore an undetermined or prolonged outbreak or to control the cleaning after CPE carrier discharge. Samples were obtained from drains (sinks and showers) and toilets (rim and brush) and were collected with sterile swabs pre-moistened with a neutraliser (DNP + thiosulfate, bioMérieux, Marcy-l’Etoile, France).

### Microbiological investigations

Samples from patients or the environment were inoculated on ChromID CARBA SMART (bioMérieux) agar and incubated at 37 °C in an aerobic atmosphere for 24 hours. For environmental samples, a pre-enrichment was also performed in a tryptic soy broth (Bio-Rad Laboratories, Marnes la Coquette, France) and incubated at 37 °C in an aerobic atmosphere for 24 hours before plating on ChromID CARBA SMART agar. Identification of suspicious colonies was performed by MALDI-TOF spectrometry (MALDI-TOF) (MALDI-TOF MS, Bruker Daltonics, Bremen, Germany).

Antimicrobial susceptibility testing was performed by the disk diffusion method on Mueller-Hinton agar (Bio-Rad Laboratories) and interpreted following the European Committee on Antimicrobial Susceptibility Testing recommendations (current version at the time of sampling).

If CPE was suspected, the isolate was tested for the presence of carbapenemase by PCR (Xpert Carba-R assay, Cepheid, Maurens-Scopont, France) or by immunochromatographic assay (RESIST-4 O.K.N.V, Coris BioConcept, Gembloux, Belgium or NG-CARBA 5, NG Biotech, Guipry, France). OXA-48-producing *C. freundii* strains from patients and from the environment were sent to the French National Reference Center (F-NRC) for sequencing.

### Whole genome sequencing

Taking into account an even distribution in time and space, 20 OXA-48-producing ST-22 *C. freundii* from the environment and 33 OXA-48-producing ST-22 *C. freundii* from patients were selected for sequencing. Short-read sequencing was performed on all strains included in the study (n = 53) using a HiSeq system (Illumina Inc, San Diego, United States) (GenBank accession numbers: BioProject PRJNA1074264). Moreover, 240 already sequenced epidemiologically unrelated OXA-48-producing *C. freundii* ST-22 from the F-NRC’s collection were included in this study. Illumina reads were assembled using shovill v1.1.0 and spades v3.14.0. MLST analysis was performed using pubMLST databases (https://pubmlst.org/) [19]. For phylogenetic single nucleotide polymorphism (SNP)-based analysis, the *C. freundiii* 124F3 (GenBank accession PRJNA1074264) strain was used as the reference genome. Metadata and phylogenetic trees were visualised and annotated using iTOL v6.5.2.

## Results

### Case characteristics

**Figure 1:**
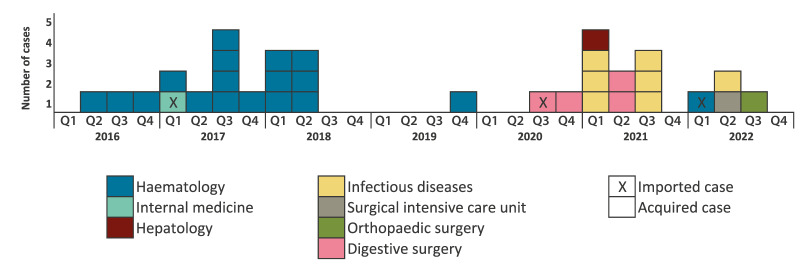
Epidemiologic curve of OXA-48-producing *Citrobacter freundii* sequence type ST-22 strains isolated from patients in hospital A, France, 2016–2022 (n = 33)

Between January 2016 and July 2022, 42,908 screening tests were performed in hospital A. In total, 33 strains of OXA-48-producing ST-22 *C. freundii* were identified from patients hospitalised in seven wards, located in three buildings (eight in building A, eight in building B and 17 in building C) (see Figure 1 and the Supplementary Table, which describes all 53 OXA-48-producing ST-22 *C. freundii* strains included in this study). The patients’ median age was 65 years (interquartile range (IQR): 49–72). Eighteen patients were male and 15 were female. Of these 33 strains, 30 were considered as acquired and three as imported. Among the 33 patients, 23 were colonised and 10 developed an infection (one bacteriaemia, seven urinary tract infections and two deep tissue infections). One patient carried a strain harbouring both *bla*_OXA-48_ and *bla*_NDM-1_ genes (149J4).

Q: quarter (Q1: 1 Jan–31 Mar, Q2: 1 Apr–30 Jun, Q3: 1 Jul–30 Sep, Q4: 1 Oct–31 Dec).

The median duration from admission to isolation of OXA-48-producing ST-22 *C. freundii* cases was 14 days (IQR: 8–29).

Between 2016 and 2019, all strains were isolated from patients in the haematology ward, except one imported strain (137I7) from a patient in the internal medicine ward, which was located in a different building to the haematology ward. Since 2020, cases were identified in five other wards (digestive surgery, infectious diseases, hepatology, surgical intensive care unit and orthopaedic surgery). According to patient-movement data, no patient from the haematology ward was hospitalised in one of those five wards after CPE acquisition. After recognising the toilets as the source of transmission in the haematology ward, and replacing all the toilet bowls and tanks in August 2018, only one patient (case) later acquired the strain in the haematology ward (241G3).

### Environmental investigations

Between 2016 and 2022, among the 37 OXA-48-producing ST-22 *C. freundii* stains isolated from the environment, 20 were selected and sent to the F-NRC for sequencing. The 20 selected strains were isolated from toilets (n = 14), toilet brushes (n = 1) and drains (shower drains n = 4, sink drain n = 1). Samples were collected from four wards (infectious diseases n = 12, haematology n = 6, hepatology n = 1 and internal medicine n = 1) located in the three buildings (two in building A, 13 in building B and five in building C) where the clinical strains were identified. All strains contained only the *bla*_OXA-48_ carbapenemase-encoding gene (Supplementary Table).

### Comparison of OXA-48-producing *Citrobacter freundii* strains

We compared 53 strains of OXA-48-producing ST-22 *C. freundii* from hospital A (20 from the environment and 33 from patients) by WGS. The SNP matrix analysis revealed one large cluster, which included 51 strains, with a maximum of 49 SNPs between two strains within this cluster. This large cluster was composed of different sub-clusters which were strictly correlated with the hospital department, suggesting strain exchanges between these different departments.

**Figure 2:**
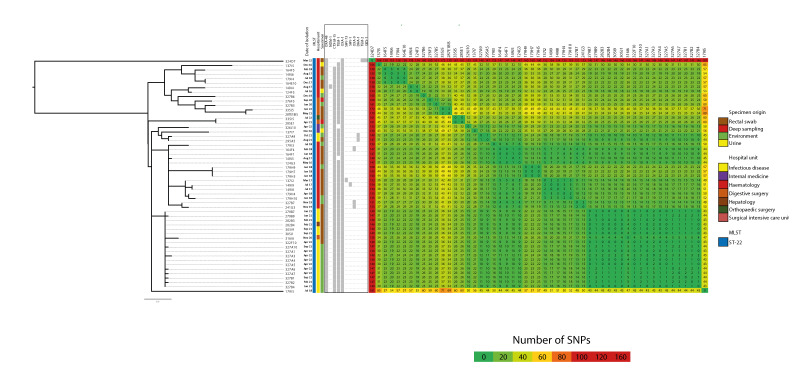
Phylogenetic tree and single nucleotide polymorphisms (SNPs) matrix of OXA-48-producing *Citrobacter freundii* ST-22 strains isolated from patients and environment in Hospital A, France, 2016–2022 (n = 53)

Two strains, 179I5 and 324D7, did not cluster with the others. The 179I5 strain exhibited differences of 43 to 71 SNPs with other strains, and the 324D7 strain exhibited differences of 146 to 181 SNPs. The 324D7 strain was considered as imported and the 179I5 strain was identified in 2018 in the environment (Figure 2). Interestingly, two other strains, 137I7 and 276F3, were also imported but seemed genetically similar to the large cluster by WGS. The patient carrying strain 276F3 had been recently hospitalised in hospital A’s digestive surgery ward before the identification of this strain, but their rectal screening was negative at that time. The patient carrying strain 137I7 was also recently hospitalised in hospital A, in the internal medicine ward, but was never screened.

**Figure 3:**
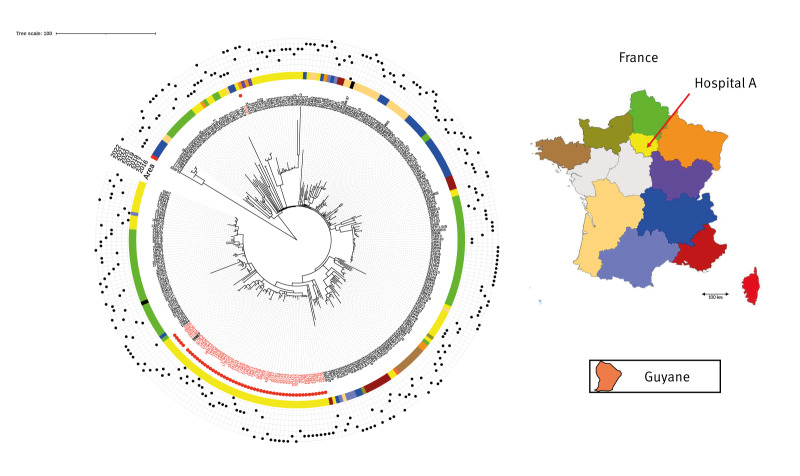
Phylogenetic tree of 293 non-duplicate OXA-48-producing *Citrobacter freundii* ST-22 strains characterised by the French National Reference Center, and including the 53 OXA-48-producing strains in our study, 2016–2022

The strains involved in this outbreak were compared with other OXA-48-producing ST-22 *C. freundii* isolated elsewhere in France (240 strains from the collection of the F-NRC for antimicrobial resistance) using WGS analysis (total n = 293) (Figure 3). All strains from patients and the environment of hospital A (except one imported case 324D7) belonged to the same cluster and were different from strains isolated in the same region or elsewhere in France. In contrast, strain 179I5, which presented with more than 50 SNPs differing from other strains isolated in hospital A, belonged to this cluster. One strain (208E7) belonged to this cluster, but was isolated in another hospital. This strain was isolated from a urine sample received at a private microbiology laboratory in Paris. The patient carrying this strain was a previously known OXA-48-producing *C. freundii* carrier who had been admitted to the hepatology ward of hospital A in 2019, but the strain isolated in hospital A at that time was not sent to the F-NRC. The SNP and phylogenetic analyses suggested a long-lasting persistence of the same clone for more than 6 years, with few genetic variations.

Red dot corresponds to strains identified in hospital A.

Black dots in the outer circle indicate the different years of isolation.

### Outbreak control measures

The investigation suggested an environmental hydric origin of OXA-48-producing *C. freundii*. Consequently, the infection prevention control team recommended multiple interventions. Screening of patients at risk for CPE carriage as previously defined (contact patients, patients from abroad or patients with a recent history of hospitalisation) was intensified (see Infection Control Measures in the Methods section). During the outbreak in the haematology ward (2016–2019), intensive toilet cleaning with descaling and bleaching was implemented, and toilets bowls and tanks with environmental CPE-positive samples were replaced by rimless toilets in 2018 [16]. Only one patient carrying the strain 241G3 in 2019 was identified in the haematology ward after taking these measures. In other wards, various interventions were implemented depending on the risk level, such as cleaning, descaling and disinfection of sinks with bleach, changing the sinks and stopping inappropriate procedures such as disposal of patients’ body fluids and patient nutrition in sinks.

## Discussion

In this study, we sequenced OXA-48-producing ST-22 *C. freundii* strains recovered in hospital A from patients as well as from toilets and drains between 2016 and 2022. The clonality of these strains was established through WGS analysis. We demonstrated that all OXA-48-producing *C. freundii* ST-22 strains in hospital A appeared to be related, except for one strain (324D7). All of them possessed fewer than 50 SNPs difference, except for one which differed by up to 71 SNPs. Since the number of SNPs depends on the alignment method and reference strain used, it is challenging to provide a precise cut-off determining clonality. However, Enterobacterales strains generally acquire 5–10 SNPs per year per genome [20,21]. Accordingly, it is not surprising to observe this high number of SNPs between two OXA-48-producing *C. freundii* ST-22 belonging to a long-term outbreak that evolved over 7 years. We also compared these strains to other OXA-48-producing ST-22 *C. freundii* isolated in France during the same period. Strains from our long-term outbreak were clearly different from strains isolated elsewhere in France. We also confirmed that the 324D7 strain recovered in hospital A was not clonally related to the long-term outbreak. These data strongly suggest the persistence of an OXA-48-producing *C. freundii* ST22 clone in the hospital environment for years, with potential acquisitions and dissemination across different buildings.

Hospital sanitary facilities (sinks, drains and toilets) have been identified as a potential source and reservoir for hospital outbreaks of CPE, including OXA-48-producing *C. freundii* [10,16]. The presence of an environmental reservoir has been associated with long-term outbreaks. Nurjadi et al. [15] described a prolonged outbreak of OXA-48-producing *Enterobacter cloacae* between 2015 and 2021 in a haematological unit, related to environmental acquisition from sinks and drains. Neidhöfer et al. [22] confirmed that sanitary facilities played a key role in maintaining a reservoir for multidrug-resistant organisms between 2014 and 2021 in oncology patients, a particular population with a high antibiotic exposure.

Experimental studies showed that toilets and drains can become contaminated by Enterobacterales through an anterograde or retrograde mechanism. Anterograde contamination may result from digestive colonised patients who use the sanitary facilities, potentially leading to the formation of bacterial biofilms. Factors that facilitate biofilm formation, such as nutrient exposure also plausibly increase the risk of CPE establishment and persistence in the wastewater environment [23,24]. Moreover, we sometimes observed non- recommended practices in hospital A, such as disposing of patients’ body fluids in sinks. Sink and drain contamination may result from a retrograde wastewater flow. Heireman et al. showed that toilet drain water may be a potential source of hospital room-to-room transmission of carbapenemase-producing *Klebsiella pneumoniae* in a burn unit [25].

Boutin et al. [26] conducted a prospective observational study on the colonisation of the hospital wastewater environment during the first year of occupancy of the intensive care unit in a new building. The authors demonstrated that cross-contamination between patients and the hospital environment can be bidirectional. Microorganisms may persist in biofilms in wastewater plumbing, then be dispersed to the surroundings by particles, droplets, or aerosol formation [27]. A recent prospective study was performed by Regev-Yochay et al. [28] between 2017 and 2019 in nine departments with systematic screening of patients and sinks. The results of WGS combined with temporal data suggested a sink-to-patient transmission for 20 patients.

In the present study, we were unable to fully understand the mechanism of transmission of OXA-48-producing ST-22 *C. freundii* between different buildings. Patient movement, multiple hospitalisations of colonised patients or contact patients between wards of different buildings or contamination of the wastewater drainage system may have promoted the dissemination of this strain.

The reason why this OXA-48-producing ST-22 *C. freundii* clone persisted and spread within the healthcare environment remains unknown and needs further investigation. This clone could be highly resistant to cleaning procedures due to biofilm formation, could have acquired tolerance to disinfectants or may have a longer survival time in wastewater. The fact that a specific clone persisted for years in hospital A and was different from other clones circulating in France points to potential genetic or phenotypic characteristics associated with environmental persistence.

Determining effective infection control measures to decontaminate environmental reservoirs and prevent biofilm formation contributes to minimising the risk of outbreaks. Previous reports have shown that eradicating drain contamination by CPE is very difficult despite bleach or hydrogen peroxide disinfection [29]. Other methods such as vapour, which combines heat and pressure, only showed short-term effectiveness. It was shown that CPE outbreaks related to an environmental reservoir have been controlled by withdrawing sinks from the ward [30]. Recently, Fucini et al. have shown that intensive care units with no sinks have lower rates of healthcare-associated infections [31] compared to those with sinks. If it is not possible to withdraw sinks, one possible intervention would be to ensure that the bed is placed at least 2 metres from the sinks in order to avoid bacterial transmission by a splashing effect. For toilets, technical measures (e.g. rimless toilets, use of cover), chemical measures (i.e. disinfection of drains) and strict hygiene precautions may help prevent cross-contamination. Since some measures, such as sink and toilet replacement, have considerable financial impact and may disrupt clinical management of patients, it is therefore crucial to ensure that strict prevention and control measures are implemented.

Our study has several limitations. First, this study was performed in a single centre, which hinders the generalisation of our results. Second, the sampling of the environment was not systematically performed following the identification of a case and all OXA-48-producing ST-22 *C. freundii* strains from the environment were not analysed by WGS. Third, contact patients’ movements after discharge were not collected. The readmission of a contact patient may explain the dissemination of this strain between different buildings.

## Conclusion

Our data suggest that a specific OXA-48-producing ST-22 *C. freundii* clone can persist in the hospital environment for years and spread through buildings, representing a risk for hospital-acquired infections and outbreaks. Reservoir management is essential to prevent further transmission in addition to strict hand hygiene.

## Supplementary Data

199000 Supplementary Material
