# Supplementary material for: Persistence of OXA-48-producing ST-22 Citrobacter freundii in patients and the hospital environment, Paris, France, 2016 to 2022
Source: Euro Surveill. 2024 Dec 5;29(49):2400262. doi: 10.2807/1560-7917.ES.2024.29.49.2400262 (PMC11650478; doi:10.2807/1560-7917.ES.2024.29.49.2400262)
Supplement: Supplementary Material [file 24-00262_JOLIVET_Supplementary_Table.pdf]

This supplementary material is hosted by *Eurosurveillance* as supporting information alongside the article "Persistence of OXA-48-producing ST-22 *Citrobacter freundii* in patients and the hospital environment, Paris, France, 2016 to 2022", on behalf of the authors, who remain responsible for the accuracy and appropriateness of the content. The same standards for ethics, copyright, attributions and permissions as for the article apply. Supplements are not edited by *Eurosurveillance* and the journal is not responsible for the maintenance of any links or email addresses provided therein.

Supplementary Table. Characteristics of OXA-48-producing *Citrobacter freundii* sequence type ST-22 strains, 2016-2022 (n = 53)

Strain reference	BioSample Accession Number	Ward	Building	Month/year of specimen collection	Infection/colonisation or environment	Delay from admission	Acquired or imported case	Carbapenemase
124G3	SAMN39855129	Haematology	A	May-16	Colonisation	37	Acquired	OXA-48
124F3	SAMN39855128	Haematology	A	July-16	Infection	25	Acquired	OXA-48
137I5	SAMN39855131	Haematology	A	December-16	Infection	63	Acquired	OXA-48
137I7	SAMN39855132	Internal medicine	B	January-17	Infection	0	Imported	OXA-48
137I2	SAMN39855130	Haematology	C	March-17	Infection	50	Acquired	OXA-48
149I8	SAMN39855133	Haematology	C	June-17	Colonisation	6	Acquired	OXA-48
149I9	SAMN39855134	Haematology	C	July-17	Colonisation	14	Acquired	OXA-48
149J4	SAMN39855135	Haematology	A	August-17	Colonisation	33	Acquired	OXA-48 + NDM-1
149J6	SAMN39855137	Haematology	C	August-17	Colonisation	11	Acquired	OXA-48
149J5	SAMN39855136	Haematology	C	August-17	Infection	32	Acquired	OXA-48
164E10	SAMN39855138	Haematology	A	December-17	Colonisation	31	Acquired	OXA-48
164F1	SAMN39855139	Haematology	A	January-18	Colonisation	14	Acquired	OXA-48
164F5	SAMN39855141	Haematology	C	February-18	Colonisation	28	Acquired	OXA-48
164F4	SAMN39855140	Haematology	A	February-18	Infection	13	Acquired	OXA-48
179H4	SAMN39855142	Haematology	C	April-18	Colonisation	5	Acquired	OXA-48
179H5	SAMN39855143	Haematology	C	June-18	Infection	42	Acquired	OXA-48
179H9	SAMN39855145	Haematology	C	June-18	Environment	-	-	OXA-48
179H10	SAMN39855146	Haematology	C	June-18	Environment	-	-	OXA-48
179H7	SAMN39855144	Haematology	A	June-18	Colonisation	17	Acquired	OXA-48
179I3	SAMN39855147	Haematology	A	July-18	Environment	-	-	OXA-48
179I5	SAMN39855149	Infectious Diseases	B	July-18	Environment	-	-	OXA-48
179I4	SAMN39855148	Haematology	A	July-18	Environment	-	-	OXA-48
241G3	SAMN39855150	Haematology	C	November-19	Colonisation	6	Acquired	OXA-48
327B7	SAMN39855178	Haematology	C	November-19	Environment	-	-	OXA-48
327B6	SAMN39855177	Haematology	C	December-19	Environment	-	-	OXA-48
327B5	SAMN39855176	Hepatology	C	January-20	Environment	-	-	OXA-48
276F3	SAMN39855151	Digestive surgery	C	September-20	Infection	1	Imported	OXA-48
316I6	SAMN39855161	Digestive surgery	C	November-20	Colonisation	16	Acquired	OXA-48
279B9	SAMN39855153	Infectious Diseases	B	January-21	Colonisation	29	Acquired	OXA-48
279B7	SAMN39855152	Infectious Diseases	B	January-21	Colonisation	8	Acquired	OXA-48
327B4	SAMN39855175	Infectious Diseases	B	January-21	Environment	-	-	OXA-48
282B3	SAMN39855154	Infectious Diseases	B	February-21	Colonisation	14	Acquired	OXA-48
282B4	SAMN39855155	Hepatology	C	February-21	Infection	6	Acquired	OXA-48
327B2	SAMN39855174	Infectious Diseases	B	February-21	Environment	-	-	OXA-48

285E1	SAMN39855156	Digestive surgery	C	April-21	Infection	11	Acquired	OXA-48
287E1	SAMN39855157	Digestive surgery	C	May-21	Colonisation	23	Acquired	OXA-48
295A5	SAMN39855158	Infectious Diseases	B	August-21	Colonisation	13	Acquired	OXA-48
305J1	SAMN39855160	Infectious Diseases	B	September-21	Colonisation	4	Acquired	OXA-48
327A10	SAMN39855172	Infectious Diseases	B	September-21	Environment	-	-	OXA-48
327B1	SAMN39855173	Infectious Diseases	B	September-21	Environment	-	-	OXA-48
305I9	SAMN39855159	Infectious Diseases	B	September-21	Colonisation	11	Acquired	OXA-48
327A9	SAMN39855171	Infectious Diseases	B	October-21	Environment	-	-	OXA-48
324D7	SAMN39855163	Haematology	C	March-22	Colonisation	0	Imported	OXA-48
322F10	SAMN39855162	Infectious Diseases	B	April-22	Colonisation	35	Acquired	OXA-48
327A3	SAMN39855166	Infectious Diseases	B	April-22	Environment	-	-	OXA-48
327A4	SAMN39855167	Infectious Diseases	B	April-22	Environment	-	-	OXA-48
327A5	SAMN39855168	Infectious Diseases	B	April-22	Environment	-	-	OXA-48
327A6	SAMN39855169	Infectious Diseases	B	April-22	Environment	-	-	OXA-48
327A7	SAMN39855170	Infectious Diseases	B	April-22	Environment	-	-	OXA-48
327A1	SAMN39855165	Infectious Diseases	B	April-22	Environment	-	-	OXA-48
326J10	SAMN39855164	Internal medicine	B	April-22	Environment	-	-	OXA-48
335J5	SAMN39855180	Surgical intensive care unit	C	June-22	Colonisation	11	Acquired	OXA-48
335I5	SAMN39855179	Orthopaedic surgery	C	July-22	Colonisation	27	Acquired	OXA-48
